# Non-Energy-Restricted Low-Carbohydrate Diet Combined with Exercise Intervention Improved Cardiometabolic Health in Overweight Chinese Females

**DOI:** 10.3390/nu11123051

**Published:** 2019-12-13

**Authors:** Shengyan Sun, Zhaowei Kong, Qingde Shi, Mingzhu Hu, Haifeng Zhang, Di Zhang, Jinlei Nie

**Affiliations:** 1 Institute of Physical Education, Huzhou University, Huzhou 313000, China; sysun@zjhu.edu.cn; 2 Faculty of Education, University of Macau, Macao 999078, China; zwkong@um.edu.mo (Z.K.); mb74811@um.edu.mo (M.H.); mb74812@um.edu.mo (D.Z.); 3 School of Health Sciences and Sports, Macao Polytechnic Institute, Macao 999078, China; qdshi@ipm.edu.mo; 4 College of Physical Education, Hebei Normal University, Shijiazhuang 050000, China; hbnuzhanghaifeng@sina.com

**Keywords:** ketogenic diet, short term, exercise, body composition, cardiorespiratory fitness, blood lipids

## Abstract

This study aimed to examine the effects of four weeks of a low-carbohydrate diet (LC) and incorporated exercise training on body composition and cardiometabolic health. Fifty-eight overweight/obese Chinese females (age: 21.2 ± 3.3 years, body mass index (BMI): 25.1 ± 2.8 kg/m^2^) were randomly assigned to the control group (CON, *n* = 15), the LC control group (LC-CON, *n* = 15), the LC and high-intensity interval training group (LC-HIIT, *n* = 15), or the LC and moderate-intensity continuous training group (LC-MICT, *n* = 13). Subjects consumed a four week LC, whereas LC-HIIT and LC-MICT received extra training 5 d/week (LC-HIIT: 10 × 6 s cycling interspersed with 9 s rest, MICT: 30 min continuous cycling at 50–60% VO_2peak_). After intervention, the three LC groups demonstrated significant reductions in body weight (−2.85 kg in LC-CON, −2.85 kg in LC-HIIT, −2.56 kg in LC-MICT, *p* < 0.001, *η*^2^ = 0.510), BMI (*p* < 0.001, *η*^2^ = 0.504) and waist-to-hip ratio (*p* < 0.001, *η*^2^ = 0.523). Groups with extra training (i.e., LC-HIIT and LC-MICT) improved VO_2peak_ by 14.8 and 17.3%, respectively. However, fasting glucose and blood lipid levels remained unchanged in all groups. Short-term LC is a useful approach to improve body composition in overweight/obese Chinese females. Incorporated exercise training has no additional effects on weight loss, but has additional benefits on cardiorespiratory fitness, and HIIT is more time efficient than the traditional MICT (2.5 min vs. 30 min).

## 1. Introduction

The obesity pandemic is a major health challenge faced by both developed and developing nations [1]. This is due to its close association with multiple comorbidities which are among the top causes of mortality, such as type 2 diabetes (T2D), cardiovascular diseases (CVDs), and cancer [2]. The beneficial health effects of weight loss are well recognized [3,4,5], and diet and exercise remain the most common strategies to fight obesity.

The conventional dietary guidelines for weight loss (i.e., low-fat, high-carbohydrate, reduced-calorie diets) [6] have been challenged, especially by supporters of low-carbohydrate diets (LCs). Emerging evidence mainly derived from western countries has clearly shown that LCs could be effective dietary strategies for weight loss and the management of several common and rare pathological conditions [7,8,9,10,11,12,13]. A meta-analysis has revealed that LCs had greater long-term effects on weight loss when compared to the traditional low-fat diets; furthermore, triglycerides, high-density lipoprotein cholesterol (HDL-C), and blood pressure values were also more favorably altered in LCs [7]. Additionally, LC-induced weight loss is generally accompanied with several positive metabolic changes, including improvements in blood pressure, glycaemic control, insulin sensitivity, and serum concentrations of C-reactive protein and certain lipids [8,9,10,11,12], which may have the potential to alleviate the features of metabolic disorder and reduce the risk factors associated with developing CVD and T2D. However, limited studies have tested whether LCs are also useful and feasible for the large Chinese overweight/obese population [11,13] given that people in China and in Western countries have distinct dietary patterns and preferences [14,15], which may lead to different levels of acceptance toward LCs. Furthermore, previous LC trials were invariably administered ad libitum without fixed daily energy intakes, or had an overemphasis on energy restriction [8,10,11,12,16,17]. Undeniably, this approach is more effective and easier to implement in public practice, where researchers are unable to provide strict controls and intensive monitors on diet as compared to the clinical setting. Yet, it is difficult to evaluate whether the beneficial cardiometabolic health outcomes resulting from these LCs should be attributed to the reduction in calorie intake or the tested dietary pattern. Therefore, with potential confounding factors being controlled, it is necessary to provide initial evidence regarding the effects of LC with a fixed or unchanged calorie intake on cardiometabolic health in the Chinese overweight/obese population.

In addition to diet, exercise is the main outlet for excessive energy, thus leading to weight reduction, although exercise and LC may work through different metabolic pathways. High-intensity interval training (HIIT) protocols have been developed in the past decade as time-saving replacements of the traditional moderate-intensity continuous training (MICT) to improve fitness and health. With less time commitment and a lower overall exercise amount, previous studies have shown that HIIT programs have the potential to improve cardiorespiratory fitness (CRF), body composition, and cardiometabolic risk factors to a similar extent as MICT programs [18,19,20,21]. Thus, combining HIIT or MICT intervention with LC may induce additional benefits on weight loss and cardiometabolic health. Furthermore, during LC intervention with minimal carbohydrate supplements, muscle glycogen stores and glycolytic enzyme activity are typically lowered if there is no preadaptation [22]. As a result, short-term LC has been reported to reduce CRF [17,23], whereas regular exercise is considered the most effective approach to improve CRF. Therefore, combined LC with HIIT or MICT may potentially compensate or even reverse the CRF-lowering effects of LC. Until now, no study has examined whether collaborate LC with time-saving HIIT or MICT have additional benefits for weight loss and cardiometabolic health, and for this reason, this is one of the research interests of the present study.

Given the above, we adopted an extremely brief HIIT protocol, involving only 1 min of intense exercise (i.e., 10 × 6 s sprint cycling interspersed with 9 s rest periods, 2.5 min in total), and combined it with LC. Our objectives were as follows: (1) To examine the efficacy of four weeks of non-energy-restricted LC on body composition and cardiometabolic health-related profiles in overweight/obese Chinese females; (2) to evaluate whether LC combined with brief HIIT or traditional MICT (i.e., continuous cycling at 50–60% peak oxygen uptake (VO_2peak_) for 30 min) would trigger additional effects on weight loss and cardiometabolic health. We hypothesized that the short-term, non-energy-restricted LC intervention alone could effectively improve body composition and cardiometabolic health in overweight/obese Chinese females, and groups incorporated with extra HIIT or MICT would have additional benefits in terms of weight loss and CRF.

## 2. Materials and Method

### 2.1. Participants

**Figure 1:**
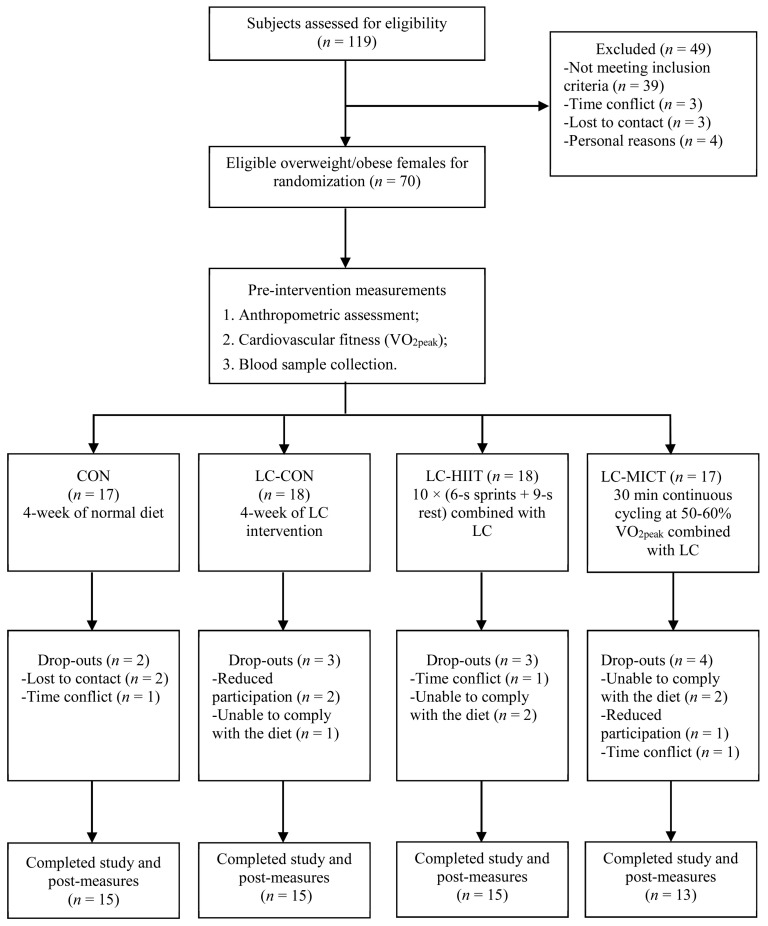
Flow-chart of the study.

This study was approved by the Research Ethics Committee of the University of Macau (RC Ref. no. MYRG2017-00199-FED) and carried out according to the Declaration of Helsinki. A power calculation was conducted to estimate the sample size using G * Power (Version 3.1). Under the assumptions of a correlation of 0.8 between pre–post intervention measurements and an effect size of 0.32 based on a meta-analysis for the primary outcome of VO_2peak_ resulting from HIIT [24], we would need to enroll 12 subjects for the HIIT group with a power of 0.8 at a significance level of 5%. When a potential dropout rate of 25% is considered, we aimed to recruit a total of 60 subjects. Public recruitment notices, including research intention and inclusion criteria, were released through the university bulletin board and e-mail to recruit eligible overweight or obese young females. The inclusion criteria were as follows: (1) between the age of 18 to 30 years old; (2) body mass index (BMI) ≥ 23 kg/m^2^ [25]; (3) maintenance of a stable body weight in the past six months (variation of less than 2 kg); (4) sedentary lifestyle, not involved in any regular or structured exercise programs in the past 6 months; and (5) healthy (no endocrine, metabolic, osteoarticular, or heart diseases). Smokers, alcoholics, and subjects who were taking prescribed medication of any kind, regularly consuming nutritional supplements, adhering to specific diet programs, suffering from respiratory problems or eating disorders were excluded. After screening, a total of 70 eligible overweight/obese young females (19–25 years old) were included. Written informed consent was obtained from all participants before they were randomly allocated to either one of the four groups, namely the normal diet control group (CON, *n* = 17), the low-carbohydrate diet control group (LC-CON, *n* = 18), the low-carbohydrate diet and HIIT group (LC-HIIT, *n* = 18), and the low-carbohydrate diet and MICT group (LC-MICT, *n* = 17). Twelve participants discontinued the intervention for different reasons, 58 participants completed the intervention and all measurements were included in the data analysis (Figure 1).

### 2.2. Experimental Design

This interventional study used a four-arm pre- and post-test comparison design. The experimental procedure was composed of a preparation period, preintervention measurements (including blood assay, anthropometric assessments, and a maximal incremental exercise test to determine VO_2peak_), a 4 week (28 days) diet intervention with or without exercise training, and postintervention measurements (Figure 1).

During the preparation period, all subjects took nutrition classes and received detailed diet instructions and individual counselling from registered dietitians. Moreover, subjects were instructed to record their normal diet and daily activities for 2 weeks before intervention as baseline, and provided self-reported menstrual phases for the past 3 months. After the preparation period, subjects undertook preintervention measures of anthropometric indices, CRF levels, and blood profiles, which finished within 3–5 days prior to intervention. For the 4 week intervention period, subjects in the CON group served as controls remaining on a normal diet, and subjects in the LC-CON group were switched to LC, but with no exercise training. Besides of changing from normal diet to LC, subjects in the LC-HIIT and LC-MICT groups performed additional supervised HIIT or MICT exercise 5 days/week. The postintervention measurements were carried out in the same way as the preintervention measurements and were completed within 72–96 h following the last intervention day. All pre–post intervention measurements were performed within the same phase of each subject’s menstrual cycle (i.e., the luteal phase), which was estimated according to the self-reported menstrual phase obtained in the preparation period.

### 2.3. Diet Intervention

Except for the CON group, subjects in the LC-CON, LC-HIIT, and LC-MICT groups were instructed to consume LC, in which carbohydrates comprised approximately 10% of the calories (~50 g/d), and about 65% of the calories were obtained from fats, with the remaining ~25% from proteins. During the preparation periods, subjects received nutrition education, and individual dietary instructions regarding how to consume meals within the targeted nutrition goals from registered dietitians. Handouts outlining the main aspects of a LC and specific lists of suitable foods, drinks, cooking recipes, and sample meals for LC were also provided to all subjects. They were free to choose food with low carbohydrate contents according to their own preferences, but were required to not change the daily energy intake as measured in the normal diet (2 weeks before the intervention). The appropriate foods for LC comprised pork, beef, fish, fowl (e.g., chickens, ducks), eggs, seafoods, cheese, all kinds of fat, oils, nonstarchy and green vegetables, nuts/seeds, water and drinks with low carbohydrate contents (e.g., green/red tea, black coffee). Although the types of fat from saturated or unsaturated sources were not restricted, subjects were encouraged to add five tablespoons of olive oil to their diet each day. Foods with high carbohydrate contents were avoided during the study period, including rice, noodles, bread, desserts, cereals, sweets, honey, beans, corns, starchy vegetables, fruits (with the exception of blueberry, lemon, and avocado), milk, yoghourt, juices, soft drinks, and alcoholic beverages.

To ensure compliance with LC, all subjects took urinary ketone assessments daily and kept 3 day food records (2 weekdays and 1 weekend day). Subjects received self-testing reagent strips (UROPAPER, Suzhou First Pharmaceutical Co. Ltd., Suzhou, China) to measure their urinary ketones daily in the early morning or after dinner [26], and the results were recorded in the offered logbook. In addition, subjects were instructed to keep 3 day food records, 2 weeks before intervention and 4 weeks during intervention. Thorough instructions on how to report food/beverage items in food records were given to the subjects in the preparation period, and they were also provided with digital scales and food measuring utensils to assure that the amounts and weights of the food/beverages ingested were documented accurately. Subjects were required to return to the laboratory each week to hand in food diaries, measure body weight, and evaluate dietary compliance. Food records were analyzed for energy intake and macronutrient content by the same dietician using the nutrition analysis and management software (NRISM, version 3.1, Beijing, China). On the basis of these results, the dieticians would offer the subjects individualized follow-up counselling and dietary suggestions. Any questions related to the experiment would be answered via WeChat, phone, e-mail, or face-to-face meetings throughout the study period.

Besides dietary and/or training intervention, subjects were required to maintain their normal daily routines and not to participate in any additional exercises throughout the study period. Their physical activities were assessed daily using validated pedometers (Yamax Digi-Walker SW-200, Tokyo, Japan). A logbook with calendar was provided to the subjects to record the results of daily urinary ketone tests, daily activities, any adverse effects or symptoms, and to document food intakes.

### 2.4. Exercise Intervention

Besides consuming LC, subjects in the LC-HIIT and LC-MICT groups took extra exercise training at the Kinesiology lab at a strictly controlled room temperature (22 °C) and humidity (50–60%). The training intervention was performed 5 days/week for a duration of 4 weeks under the supervision of two trained research assistants. For the LC-HIIT group, subjects performed intermittent sprint cycling on a cycle ergometer (Monark 894E, Varberg, Sweden). Each training session comprised 10 bouts of 6 s cycling sprints followed by 9 s passive recoveries (a total of 2.5 min/session). Subjects pedaled as fast as possible during the 6 s sprint phases and rested on the seat during the 9 s recovery periods. The initial workload was set at 1 kg. If the cycling speed of all exercise bouts exceeded 100 rpm for two consecutive training sessions, the workload was increased by 0.5 kg until arriving at a workload equivalent to 5% of the subject’s body weight. Ratings of perceived exertion (RPE, Borg 6–20 scale) [27] were recorded before and immediately after the 5th and 10th exercise bouts. The power output and heart rate (HR) for each exercise bout were recorded automatically by the Monark Anaerobic Test software. For the LC-MICT group, subjects cycled continuously for 30 min on a Monark cycle ergometer (839E, Varberg, Sweden). The exercise intensity was 50% of VO_2peak_ for the first 10 training sessions and increased to 60% of VO_2peak_ for the last 10 training sessions. The cycling speed was maintained at 50 ± 5 rpm throughout each training session. RPE and HR were recorded for every 5 min.

To assess the exercise energy expenditure of HIIT and MICT, oxygen uptake (VO_2_) during exercise was measured breath-by-breath using a gas analyzer (Vmax Encore, CareFusion Corp., San Diego, CA, USA) in the 1st, 10th, and 20th training sessions. The exercise energy expenditure was computed as 4.8 (Kcal·L^−1^) × VO_2_ (L·min^−1^) × exercise time (min), where VO_2_ was the mean oxygen consumption value throughout the training session, and the exercise time was 2.5 min for HIIT and 30 min for MICT.

### 2.5. Pre- and Post-Intervention Measurements

#### 2.5.1. Blood Profiles

Blood samples were collected before and 72 h after the last intervention by a certificated nurse. Strenuous physical activity, caffeine, and alcohol were confined 48 h prior to blood sampling. The subjects were required to arrive at the laboratory before 7:30 a.m. after a 12 h overnight fast, and 5 mL blood samples were extracted from the cubital vein using serum separation tubes. The blood samples were set aside clotting for 1 h at room temperature and subsequently centrifuged at 3000 rpm for 5 min, separated for serum and frozen immediately at −80 °C for further analysis.

An automatic biochemical analyzer (Olympus AU400, Olympus, Tokyo, Japan) was used to assay blood glucose, total cholesterol (CHOL), total triglyceride (TG), HDL-C, and low-density lipoprotein cholesterol (LDL-C) profiles. The coefficients of variations for intra-assay and inter-assay were all below 3%. All blood samples were assayed in standard procedures in accordance with the manufacturer’s instructions (KingMed Diagnostics Co., Ltd., Guangzhou, China).

#### 2.5.2. Anthropometric Assessments

After blood sampling, each subject underwent anthropometric assessments in the same morning. Height and weight were measured via standard methods (without shoes and in light clothing), using a wall-mounted stadiometer and an electronic scale, and the values were accurate to 0.1 cm and 0.1 kg, respectively. The BMI (in kg/m^2^) was calculated as weight (kg) divided by height squared (m^2^). Waist circumference (WC) was measured at the level midway between the rib cage and the iliac crest when the participant was gently breathing out. Hip circumference (HC) was determined as the maximum circumference over the buttocks; the WC and HC values were recorded to the nearest 0.5 cm. Waist-to-hip ratio (WHR) was calculated as WHR = WC/HC (in cm). The pre–post intervention anthropometric assessments were performed by the same research staff.

#### 2.5.3. Maximal Incremental Exercise Test

All subjects undertook two maximal incremental exercise tests before and 72 h after the last intervention session to determine their VO_2peak_ and CRF levels. After warming-up at 25 W for 3 min, subjects began to pedal on a computer-controlled cycle ergometer (Monark 839E, Varberg, Sweden) with the starting workload of 50 W. The workload during exercise would be increased by 25 W for every 3 min until the subjects reached their endurance limit, followed by a 3 min recovery period at 25 W. Throughout the test, the cycling cadence was maintained at 60 ± 5 rpm, and the respiratory gases were continuously analyzed by the Vmax Encore System (CareFusion Corp., San Diego, CA, USA). The highest oxygen consumption value averaged over 15 s of the final stage was considered as the VO_2peak_ [28].

### 2.6. Statistical Analysis

The PASW software (Release 22.0; IBM, New York, NY, USA) was used to perform statistical analyses. Prior to the main statistical analyses, the normality assumption of outcome variables was confirmed using the Shapiro–Wilk test. Analysis of covariance (ANCOVA) with the preintervention values as covariate was used to examine the group differences of main outcome variables among the four groups. One-way ANOVA tests were computed to assess the differences of changes in outcome variables after intervention and the daily activities, energy intake and macronutrient proportion among the four groups. Independent samples *T*-test was used to detect the differences of exercise training data between LC-HIIT and LC-MICT. Partial *η*^2^ values were used to assess effect sizes of the main and interaction effects; *η*^2^ was considered small if <0.06 and large if >0.14 [29]. Cohen’s *d* values were also calculated to evaluate the effect sizes for the difference between variables, which was considered small when *d* was 0.2–0.3, medium when *d* was around 0.5 and large when *d* > 0.8 [30]. The outcome variables are shown as means (standard deviations, SDs), and the statistical significance level was set at *p* < 0.05.

## 3. Results

### 3.1. Compliance, Diet Compositions, and Daily Physical Activities

Approximately 3 days after LC consumption, subjects were able to detect ketone bodies in the urine. When the data of the three initial transition days were excluded, urinary ketosis was detected on 97.6 ± 4.5%, 96.2 ± 8.3%, and 96.9 ± 6.0% of the days in the LC-CON, LC-HIIT, and LC-MICT groups, respectively, whereas the percentage was only 2.6 ± 9.2% in the CON group, indicating that subjects in LC dietary groups had good compliance with LC.

**Figure 2:**
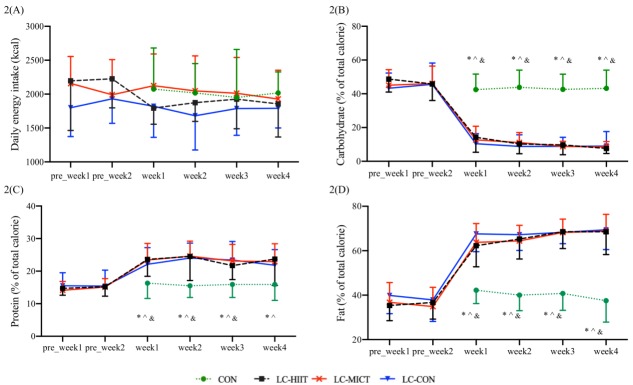
Daily dietary intake (**A**), and proportions of carbohydrate (**B**), protein (**C**), and fat (**D**) in energy intake before and during the intervention. * *p* < 0.05 compared to LC-HIIT, ^ *p* < 0.05 compared to LC-MICT, and *p* < 0.05 compared to LC-CON.

At baseline, the reported normal dietary energy intake and nutrient compositions were similar among the three LC groups. Average energy intake in the habitual diet was 2057 ± 437 kcal, with carbohydrates, proteins, and fats accounting for 45.9 ± 8.0% (236 ± 59 g), 15.0 ± 2.7% (77 ± 23 g), and 36.9 ± 6.9% (84 ± 25 g) of the total energy intake, respectively. During the intervention period, mean energy intake and the proportion of energy intake derived from carbohydrate, protein, and fat were 1776 ± 284 kcal, 9.3 ± 5.5% (41 ± 24 g), 22.8 ± 3.2% (100 ± 21 g), and 68.1 ± 4.6% (135 ± 25 g) in the LC-CON group; 1871 ± 246 kcal, 10.5 ± 3.2% (49 ± 17 g), 23.3 ± 4.7% (109 ± 28 g), and 66.1 ± 6.1% (137 ± 17 g) in the LC-HIIT group; and 2028 ± 284 kcal, 10.3 ± 2.9% (52 ± 15 g), 23.5 ± 3.2% (119 ± 23 g), and 66.3 ± 3.1% (149 ± 23 g) in the LC-MICT group; whereas the CON group remained at 1990 ± 345 kcal, 43.1 ± 7.9% (216 ± 48 g), 15.9 ± 3.6% (78 ± 23 g), and 40.2 ± 5.7% (88 ± 20 g) (Figure 2A–D). Daily energy intakes were not changed from the habitual diet in all groups (*p* > 0.05), whereas macronutrient intakes were changed significantly, with higher protein (*p* < 0.05) and fat (*p* < 0.01) consumptions and lower carbohydrate consumption (*p* < 0.01) as compared to the normal diet and the CON group (data are presented in Supplementary Materials Table S1).

There were no statistical differences in terms of daily physical activities among the CON group (7770–8867 steps), the LC-HIIT group (7779–9156 steps), the LC-MICT group (8028–9317 steps), and the LC-CON group (7694–8852 steps) at baseline or any subsequent time point (data are presented in Supplementary Materials Table S2).

### 3.2. Training Data

For the two groups with additional exercise training (i.e., LC-HIIT and LC-MICT), the total training time spent in MICT (600 min) was 12 times that spent in HIIT (50 min). Similarly, the exercise energy expenditure of MICT (148.6 ± 15.5 kcal/session) was significantly larger than that of HIIT (17.6 ± 2.2 kcal/session, *p* < 0.01). The exercise intensity corresponded to 86.8 ± 9.5% and 59.5 ± 6.6% of VO_2peak_ for the LC-HIIT group and the LC-MICT group, respectively. During exercise training, subjects in the LC-HIIT group maintained a higher mean HR level (146 ± 5 bpm) than those of the LC-MICT group (139 ± 8 bpm, *p* < 0.01), and HIIT training was perceived to be harder than MICT training, as reflected by the self-reported RPE value (14 ± 1 in the LC-HIIT group vs. 11 ± 1 in the LC-MICT group, *p* < 0.01) (Table 1).

### 3.3. Anthropometric Parameters

After intervention, all three LC groups (i.e., LC-CON, LC-HIIT, and LC-MICT) experienced remarkable reductions in body weight (*p* < 0.01, *η*^2^ = 0.510) and BMI (*p* < 0.01, *η*^2^ = 0.504), whereas no change in body weight or BMI was found in the CON group (Table 2). On post-test, body weight was reduced by 2.85 ± 1.5, 2.85 ± 1.1, and 2.56 ± 1.3 kg in the LC-CON group, LC-HIIT group, and LC-MICT group, respectively (Table 3). Correspondingly, postintervention BMI was decreased by 1.09 ± 0.56, 1.07 ± 0.41, and 0.98 ± 0.49 kg/m^2^ in the groups of LC-CON, LC-HIIT, and LC-MICT, respectively (Table 3). The LC intervention also significantly lowered WC (*p* < 0.01, *η*^2^ = 0.523), HC (*p* < 0.01, *η*^2^ = 0.338), and WHR (*p* < 0.01, *η*^2^ = 0.313) in all three LC groups, while it remained unchanged in the CON group (Table 2). The decrements of body weight, BMI, WC, HC, and WHR of the three LC groups were significantly more than that of the CON group (*p* < 0.05); however, there was no group difference among the three groups consuming LC (*p* > 0.05, Table 3).

### 3.4. Cardiorespiratory Fitness Levels

We observed that only the two groups with extra training (i.e., LC-HIIT, LC-MICT) improved CRF, as reflected by VO_2peak_, which remained unchanged in the control groups of CON and LC-CON. The VO_2peak_ was increased by 14.8% (+3.40 ± 2.22 mL·min^−1^·kg^−1^) and 17.3% (+3.70 ± 3.00 mL·min^−1^·kg^−1^) in the LC-HIIT group and LC-MICT group, respectively, which was significantly larger than that of the CON group (−0.21 ± 2.46 mL·min^−1^·kg^−1^) and the LC-CON group (−0.37 ± 3.75 mL·min^−1^·kg^−1^, *p* < 0.05, *η*^2^ = 0.193) (Table 3).

### 3.5. Blood Lipids and Fasting Glucose

Blood lipid and fasting glucose levels did not differ among the four groups at baseline and postintervention (*p* > 0.05, Table 2 and Table 3).

### 3.6. Adverse Events

During the study period, we received a total of 20 complaints from 16 subjects in the LC-CON (seven complaints), LC-HIIT (seven complaints) and LC-MICT (six complaints) groups, whereas no feedback of adverse events was offered by subjects in the CON group. Luckily, none of them were adverse clinical events. The main symptoms experienced were similar to those reported in previous studies [8,12,17], including fatigue (7, or 35%), reduced appetite (4, or 20%), constipation (6, or 30%), diarrhea (2, or 10%), and headache (1, or 5%).

## 4. Discussion

This study proved that using a four week non-calorie-restricted LC intervention, with or without extra exercise, is able to rapidly improve a series of anthropometric profiles in overweight/obese Chinese young females. In particular, we observed significant reductions in body weight, BMI, WC, HC, and WHR, which are related to the risks of developing CVD and T2D. Moreover, this study firstly combined a brief HIIT or traditional MICT with LC. Although the collaborated training had no synergistic effects on anthropometric and blood parameters, groups with extra training (i.e., LC-HIIT and LC-MICT) showed additional improvements in CRF as compared to LC intervention alone (i.e., LC-CON). Most importantly, the LC-HIIT group obtained the same improvement in CRF to that of the LC-MICT group in less time and with a lower training amount. Thus, LC in combination with HIIT may represent an optimal choice for weight loss and CRF improvement.

Unlike the majority of previous studies which evaluated the effects of LC dietary patterns when delivered with ad libitum eating patterns or with energy restriction, this study was designed to assess whether short-term LC is also effective in the overweight/obese Chinese population without changing their habitual calorie intake. The present data showed that with professional support, the overweight/obese Chinese females in all three LC groups lost more than 2.5 kg of body weight, or ~1 unit of BMI following four weeks of LC administration. The amount of weight reduction was similar to that of other non-energy-restricted studies which reported ~2.0 kg weight losses in western adults after adopting LC for six weeks [17,31], but smaller than ~8.0 kg weight reductions in obese Chinese adults following eight weeks [11] and 12 weeks [13] of calorie-reduced LCs. With unchanged calorie intake, the weight-loss effects of the present study should be interpreted as resulting from changes in macronutrient composition. In addition to weight loss, significant reductions in WC and WHR were also detected in the overweight/obese females. As surrogate markers of visceral adipose tissue [32], decrements in WC and WHR are meaningful to reduce cardiac and metabolic risks [33]. The plausible mechanisms responsible for body composition improvements following non-caloric-restricted LC may include a decrease in lipogenesis and an increase in lipolysis, as regulated by LC-induced changes in insulin and other hormones [34], as well as an increased reliance on gluconeogenesis for energy production under high-fat, low-carbohydrate conditions, which is an energy-demanding process costing ~400–600 kcal/day of extra energy [35,36]. Nonetheless, we found no collaborative weight-loss effects of exercise training in either the LC-HIIT group or the LC-MICT group. This finding was consistent with our previous observation that short-term exercise training was unlikely to result in weight loss [37,38].

Many studies, varying in intervention length, have shown that LC dietary approaches were beneficial to blood glucose and lipid regulation [8,9,11,12,31], whereas several other studies have reported elevated LDL-C and/or TC levels in response to LC [16,17,39]. However, the present study did not detect any changes in circulating levels of glucose and blood lipids, even in the groups with extra training. The reasons for the diverse results of blood profiles among studies remain unknown, but may include differences in LC dietary approaches, intervention durations, subjects’ health status and the baseline blood glucose and lipid values. The current study illustrated that the four week LC had no adverse effects on blood profiles in the overweight/obese, otherwise healthy females with an initially normal baseline level.

The CRF, as measured during incremental exercise to exhaustion, is a strong and independent marker for CVD and all-cause mortality [40,41]. However, with an insufficient energy supply, the LC-induced changes in energy metabolism have been reported to subsequently impair CRF [17,23]. In contrast, the present study found an unchanged CRF level in the LC-CON group, which was supported by other studies [39,42]. These findings indicate that LC dietary interventions are unlikely to improve CRF, if not reduce it. Therefore, the significant increments of CRF observed in the LC-HIIT group and the LC-MICT group could be attributed to the additional exercise training, despite having no additional effects on weight loss and other cardiometabolic profiles. As revealed by meta-analysis, every 1 metabolic equivalent (MET) increment in CRF was associated with a 13% and 15% risk reduction in all-cause and CVD mortality, respectively [40]. Thus, the ~3.5 mL·min^−1^·kg^−1^ (corresponding to ~1 MET) increment of CRF yielded in the LC-HIIT and LC-MICT groups may lower the risks of all-cause and CVD mortality with important clinical significance. Surprisingly, the brief HIIT improved CRF to the same extent as MICT, but with only 1/12 of the time commitment and less than 1/8 of the exercise energy expenditure, indicating that HIIT is more efficient than the traditional MICT in improving CRF.

Nonetheless, our study has a few limitations. First, subjects in the CON group only recorded their normal diet and physical activity data for four weeks during the study period. From the perspective of study design, it would be better to evaluate their baseline values two weeks prior to intervention, just like the other three groups. Second, given that body composition was not assessed via dual-energy X-ray absorptiometry (DXA) or other techniques, we are unable to specify which composition (e.g., body water, fat mass) was associated with the main weight loss induced by LC. Third, considering that the Chinese population is scarcely involved in LC studies, with an unknown acceptance level, we chose a short-term intervention to start with. Despite the successful weight loss, the longer effects of LC on weight loss and weight maintenance are unclear. In this sense, more randomized controlled trials with longer periods are expected to reveal the long-term weight-loss effects and the underlying mechanisms of LC in the overweight/obese Chinese population.

## 5. Conclusions

Collectively, the four week non-energy-restricted LC program led to significant weight loss and visceral fat mass loss without adverse clinical events, indicating that the LC dietary patterns could also be effective and feasible for the overweight/obese Chinese population. The strength of this study is combined LC with extra exercise training, and we report, for the first time, that the collaborated HIIT and MICT would result in a marked improvement in CRF, which cannot be attained through LC intervention, despite having no additional effects on weight loss. Such an increment in CRF could be clinically beneficial to the overweight/obese population who are at a higher risk of developing CVD and T2D [2]. Given that the same CRF increment was yielded within much less time when using HIIT (2.5 min/session in HIIT vs. 30 min/session in MICT), we believe that combining LC with brief HIIT is able to trigger additional cardiometabolic health benefits beyond weight reduction, thus representing a better and time-efficient combination for health promotion in people with insufficient time.

## Figures and Tables

**Table 1:** Training data during exercise intervention.

	LC-HIIT	LC-MICT
Weekly training time (min)	12.5	150
Total training time (min)	50	600
Energy expenditure (kcal)	17.6 (2.2) **	148.6 (15.5)
Training power (W)	248.7 (34.1) **	53.8 (9.7)
Intensity (% VO_2peak_)	86.8 (9.5) **	59.5 (6.6)
Training HR (bpm)	146 (5) **	139 (8)
Training HR/HRmax (%)	81.7 (3.5) **	74.9 (3.1)
Training RPE	14 (1) **	11 (1)

All values are presented as means (standard deviations). LC-HIIT: low-carbohydrate diet combined with high-intensity interval training, LC-MICT: low-carbohydrate diet combined with moderate- intensity continuous training, HR: heart rate, RPE: ratings of perceived exertion. Comparison with LC-MICT at ** *p* < 0.01.

**Table 2:** Outcome variables before and after four weeks of intervention.

	CON (*n* = 15)		LC-CON (*n* = 15)		LC-HIIT (*n* = 15)		LC-MICT (*n* = 13)		Group Effect	
	Pre	Post	Pre	Post	Pre	Post	Pre	Post	*p*	*Partial η^2^*
Age (y)	21.6 (3.9)		20.9 (3.7)		20.8 (2.7)		21.5 (3.1)			
Height (cm)	163 (5.6)		161.3 (4.7)		162.9 (6.2)		160.9 (4.3)			
Weight (kg)	66.0 (10.2)	66.1 (10.8)	65.1 (7.3)	62.3 (6.7) *	67.9 (10.3)	65.0 (9.7) *	64.5 (6.4)	61.9 (6.0) *	0.000	0.510
BMI (kg·m^−2^)	24.8 (3.2)	24.8 (3.4)	25.0 (2.9)	24.0 (2.7) *	25.5 (3.1)	24.4 (2.9) *	24.9 (1.9)	23.9 (1.8) *	0.000	0.504
WC (cm)	77.7 (9.0)	78.5 (9.2)	78.0 (6.9)	74.0 (6.2) *	79.5 (9.4)	75.7 (9.5) *	75.5 (6.3)	71.2 (4.8) *	0.000	0.523
HC (cm)	100.5 (6.1)	100.4 (6.9)	100.7 (3.9)	98.2 (3.9) *	100.3 (4.8)	98.5 (5.3) *	101.0 (5.1)	97.7 (5.3) *	0.000	0.338
WHR	0.77 (0.1)	0.78 (0.1)	0.77 (0.0)	0.75 (0.0) *	0.79 (0.1)	0.77 (0.1) *	0.75 (0.0)	0.73 (0.0) *	0.000	0.313
VO_2peak_ (mL·min^−1^·kg^−1^)	25.6 (4.0)	25.4 (4.2)	25.2 (4.3)	24.8 (2.4)	23.3 (2.5)	26.7 (3.4) *^#^	23.4 (4.4)	27.1 (3.8) *^#^	0.009	0.193
FG (mmol·L^−1^)	4.6 (0.5)	4.7 (0.3)	4.7 (0.4)	4.6 (0.4)	4.9 (0.5)	4.7 (0.6)	4.8 (0.3)	4.8 (0.3)	0.307	0.065
CHOL (mmol·L^−1^)	4.6 (0.8)	4.9 (0.7)	5.1 (1.0)	6.1 (1.3)	4.5 (0.7)	5.3 (1.0)	4.5 (0.6)	5.1 (1.0)	0.087	0.115
HDL-C (mmol·L^−1^)	1.6 (0.4)	1.6 (0.4)	1.7 (0.3)	1.7 (0.3)	1.5 (0.3)	1.5 (0.4)	1.5 (0.3)	1.7 (0.4)	0.470	0.046
LDL-C (mmol·L^−1^)	2.7 (0.7)	3.0 (0.6)	3.3 (1.0)	4.2 (1.2)	2.8 (0.6)	3.6 (0.8)	2.8 (0.5)	3.4 (0.9)	0.084	0.117
TG (mmol·L^−1^)	1.1 (1.0)	1.1 (1.0)	1.0 (0.5)	1.1 (0.6)	1.2 (0.4)	0.9 (0.3)	0.9 (0.4)	0.8 (0.2)	0.229	0.077

Outcome variables are presented as means (standard deviations). Significant difference from CON at * *p* < 0.01, significant difference from LC-CON at # *p* < 0.01. Partial *η*^2^ value for effect size (ES). CON: control group, LC-CON: low-carbohydrate diet control group, LC-HIIT: low-carbohydrate diet combined with high-intensity interval training, LC-MICT: low-carbohydrate diet combined with moderate-intensity continuous training, BMI: body mass index, WC: waist circumference, HC: hip circumference, WHR: waist-to-hip ratio, VO_2peak_: peak oxygen uptake, FG: fasting glucose, CHOL: total cholesterol, HDL-C: high-intensity lipoprotein cholesterol, LDL-C: low-intensity lipoprotein cholesterol, TG: triglycerides.

**Table 3:** Changes in outcome variables after intervention.

	CON (*n* = 15)	LC-CON (*n* = 15)	LC-HIIT (*n* = 15)	LC-MICT (*n* = 13)	ES (*d*)		
					LC-CON	LC-HIIT	LC-MICT
∆ Weight (kg)	0.09 (1.25)	−2.85 (1.50) **	−2.85 (1.12) **	−2.56 (1.31) **	2.12	2.48	2.07
∆ BMI (kg·m^−2^)	0.02 (0.48)	−1.09 (0.56) **	−1.07 (0.41) **	−0.98 (0.49) **	2.14	2.45	2.08
∆ WC (cm)	0.8 (1.47)	−4.02 (2.36) **	−3.81 (1.98) **	−4.36 (2.87) **	2.45	2.65	2.32
∆ HC (cm)	−0.13 (1.52)	−2.51 (1.93) **	−1.83 (1.78) *	−3.27 (1.40) **	1.37	1.03	2.14
∆ WHR	0.01 (0.01)	−0.02 (0.03) **	−0.02 (0.02) **	−0.02 (0.03) **	1.35	1.96	1.41
∆ VO_2peak_ (ml·min^−1^·kg^−1^)	−0.21 (2.46)	−0.37 (3.75)	3.41 (2.22) **^#^	3.67 (3.00) **^#^	0.05	1.55	1.43
∆ VO_2peak_%	−0.62 (9.26)	−0.37 (14.06)	14.76 (9.62) **^#^	17.32 (14.24) **^#^	0.08	1.63	1.52
∆ FG (mmol·L^−1^)	0.11 (0.31)	−0.13 (0.41)	−0.21 (0.42)	−0.07 (0.30)	0.68	0.86	0.60
∆ CHOL (mmol·L^−1^)	0.31 (0.64)	0.98 (1.07)	0.77 (0.84)	0.61 (0.63)	0.75	0.62	0.47
∆ HDL-C (mmol·L^−1^)	0.01 (0.17)	0.04 (0.25)	0.09 (0.18)	0.13 (0.16)	0.14	0.48	0.70
∆ LDL-C (mmol·L^−1^)	0.31 (0.52)	0.89 (0.98)	0.78 (0.72)	0.58 (0.54)	0.74	0.74	0.50
∆ TG (mmol·L^−1^)	−0.03 (0.24)	0.09 (0.65)	−0.23 (0.43)	−0.16 (0.28)	0.25	0.55	0.49

Outcome variables are presented as mean ± standard deviation. Significant difference from CON at * *p* < 0.05, ** *p* < 0.01, significant difference from LC-CON at # *p* < 0.01. Cohen’s *d* value for effect size (ES) when compared to the CON group. Delta (∆): change from pre–post intervention, CON: control group, LC-CON: low-carbohydrate diet control group, LC-HIIT: low-carbohydrate diet combined with high-intensity interval training, LC-MICT: low-carbohydrate diet combined with moderate-intensity continuous training, BMI: body mass index, WC: waist circumference, HC: hip circumference, WHR: waist-to-hip ratio, VO_2peak_: peak oxygen uptake, FG: fasting glucose, CHOL: total cholesterol, HDL-C: high-intensity lipoprotein cholesterol, LDL-C: low-intensity lipoprotein cholesterol, TG: triglycerides.

## Supplementary Materials

The following are available online at https://www.mdpi.com/2072-6643/11/12/3051/s1, Table S1: Dietary energy intake and nutrient compositions before and during intervention, Table S2: Daily physical activities before and during intervention.
