# Supplementary material for: Non-Energy-Restricted Low-Carbohydrate Diet Combined with Exercise Intervention Improved Cardiometabolic Health in Overweight Chinese Females
Source: Nutrients. 2019 Dec 13;11(12):3051. doi: 10.3390/nu11123051 (PMC6950598; doi:10.3390/nu11123051)
Supplement: Supplementary file 1 [file nutrients-11-03051-s001.pdf]

Table S1. Dietary energy intake and nutrient compositions before and during intervention.

	Pre_Week1	Pre_Week2	Week1	Week2	Week3	Week4
Energy intake (kcal)						
CON			2074 (606.1)	2017 (433.4)	1951 (708.0)	2018 (309.4)
LC-HIIT	2195 (730.1)	2225 (428.0)	1794 (238.0)	1874 (276.3)	1926 (436.2)	1855 (487.3)
LC-MICT	2157 (399.1)	1990 (519.2)	2124 (469.0)	2047 (517.1)	2013 (528.2)	1927 (425.1)
LC-CON	1799 (424.5)	1931 (362.6)	1820 (458.2)	1680 (505.0)	1787 (393.9)	1790 (288.6)
Carbohydrate (% of energy intake)						
CON			42.5 (9.2) *^&	43.8 (10.2) *^&	42.6 (9.0) *^&	43.2 (10.8) *^&
LC-HIIT	48.7 (7.7)	45.8 (9.8)	14.2 (8.8)	10.3 (5.8)	9.7 (5.8)	7.6 (3.0)
LC-MICT	45.1 (9.2)	46.1 (10.3)	12.7 (8.1)	11.1 (5.9)	8.8 (3.0)	8.5 (3.2)
LC-CON	43.3 (9.0)	45.6 (12.6)	10.4 (6.0)	8.8 (6.9)	8.8 (5.4)	9.0 (8.6)
Fat (% of energy intake)						
CON			42.2 (6.0) *^&	40.0 (7.0) *^&	40.8 (7.6) *^&	37.5 (9.6) *^
LC-HIIT	35.4 (6.9)	36.7 (7.5)	62.3 (9.5)	65.3 (9.0)	68.6 (7.6)	68.5 (10.2)
LC-MICT	36.8 (8.9)	34.9 (8.6)	63.8 (8.4)	64.4 (7.0)	68.2 (6.0)	68.8 (7.6)
LC-CON	39.9 (8.2)	37.9 (9.7)	67.6 (8.0)	67.2 (7.1)	68.3 (5.1)	69.4 (8.9)
Protein (% of energy intake)						
CON			16.3 (4.7) *^&	15.5 (3.6) *^&	15.9 (4.0) *^&	15.9 (4.9) *^&
LC-HIIT	14.7 (2.1)	15.2 (2.9)	23.6 (5.2)	24.5 (7.4)	21.7 (4.3)	23.7 (8.1)
LC-MICT	14.1 (2.7)	15.1 (2.6)	23.4 (5.1)	24.6 (4.6)	23.1 (5.1)	22.9 (5.5)
LC-CON	15.5 (4.0)	15.4 (4.9)	22.1 (5.1)	24.0 (4.6)	23.4 (5.7)	21.8 (4.8)
Carbohydrate (in g)						
CON			223.0 (86.1) *^&	221.4 (68.0) *^&	209.9 (99.1) *^&	216.9 (58.3) *^&
LC-HIIT	265.9 (96.2)	255.5 (71.7)	62.6 (39.0)	48.4 (27.8)	49.0 (36.0)	35.7 (17.1)
LC-MICT	240.8 (58.1)	232.2 (86.3)	68.4 (46.0)	55.1 (29.5)	43.3 (14.8)	40.8 (18.5)
LC-CON	196.1 (67.2)	214.8 (53.9)	47.7 (30.6)	36.5 (27.2)	38.7 (23.8)	40.2 (20.2)
Fat (in g)						
CON			96.6 (28.5) *^&	89.5 (23.1) *^&	87.1 (29.9) *^&	84.7 (27.0) *^&
LC-HIIT	87.0 (36.6)	90.5 (24.3)	124.4 (25.9)	135.7 (26.5)	145.9 (35.0)	139.9 (37.5)
LC-MICT	89.2 (31.1)	75.5 (21.2)	151.1 (41.6)	146.8 (39.8)	153.2 (43.7)	146.5 (30.5)
LC-CON	78.2 (18.6)	83.4 (31.8)	137.4 (41.5)	126.1 (41.9)	136.5 (36.8)	138.0 (30.0)
Protein (in g)						
CON		.	82.3 (25.6) *^	77.9 (22.3) *^	78.0 (31.7) *^&	79.9 (23.6) *^
LC-HIIT	81.3 (29.7)	84.1 (21.7)	106.3 (29.4)	115.4 (30.1)	103.9 (25.9)	112.04 (28.0)
LC-MICT	75.6 (19.0)	74.5 (21.9)	121.8 (29.2)	126.6 (33.7)	115.8 (38.4)	112.1 (34.8)
LC-CON	71.6 (29.6)	74.3 (25.8)	98.8 (27.6)	99.6 (33.0)	102.8 (27.8)	97.6 (25.8)

Outcome variables are presented as means (standard deviations). CON: control group, LC-CON: low-carbohydrate diet control group, LC-HIIT: low-carbohydrate diet combined with high-intensity interval training, LC-MICT: low-carbohydrate diet combined with moderate-intensity continuous training.* $p < 0.05$ compared to LC-HIIT, ^ $p < 0.05$ compared to LC-MICT, & $p < 0.05$ compared to LC-CON.

Table 2. Daily physical activities before and during intervention.

Steps	CON	LC-HIIT	LC-MICT	LC-CON
pre_week1		7838 (3282)	8182 (2548)	8852 (1846)
pre_week2		7779 (2648)	8028 (1401)	7822 (1952)
week1	7770 (3083)	8400 (1775)	9317 (1780)	8029 (2012)
week2	7559 (3187)	8341 (2162)	9297 (2323)	8312 (3061)
week3	8867 (2165)	9156 (1793)	8461 (1934)	7694 (2978)
week4	8038 (2174)	8147 (2092)	8422 (2331)	7483 (1725)

Outcome variables are presented as means (standard deviations). CON: control group, LC-CON: low-carbohydrate diet control group, LC-HIIT: low-carbohydrate diet combined with high-intensity interval training, LC-MICT: low-carbohydrate diet combined with moderate-intensity continuous training.
